# Accuracy and complication rates of external ventricular drain placement with twist drill and bolt system versus standard trephine and tunnelation: a retrospective population-based study

**DOI:** 10.1007/s00701-020-04247-3

**Published:** 2020-02-04

**Authors:** Nadia Mansoor, Mattis A. Madsbu, Nina M. Mansoor, Andreas N. Trønnes, Oddrun A. Fredriksli, Øyvind Salvesen, Asgeir S. Jakola, Ole Solheim, Sasha Gulati

**Affiliations:** 1 Department of Neurosurgery, St. Olavs University Hospital, Trondheim, Norway; 2 Department of Neuromedicine and Movement Science, Norwegian University of Science and Technology (NTNU), Trondheim, Norway; 3 Department of Radiology, Kings College Hospital NHS Foundation Trust, London, UK; 4 Department of Public Health and General Practice, Norwegian University of Science and Technology (NTNU), Trondheim, Norway; 5 Department of Neurosurgery, Sahlgrenska University Hospital, Gothenburg, Sweden; 6 Institute of Neuroscience and Physiology, Sahlgrenska Academy, Gothenburg, Sweden

**Keywords:** Bolt drain, External ventricular drain, Hydrocephalus, Kakarla score, Tunnelated drain, Neurosurgery

## Abstract

**Background:**

An external ventricular drain (EVD) is typically indicated in the presence of hydrocephalus and increased intracranial pressure (ICP). Procedural challenges have prompted the development of different methods to improve accuracy, safety, and logistics.

**Objectives:**

EVD placement and complications rates were compared using two surgical techniques; the standard method (using a 14-mm trephine burrhole with the EVD tunnelated through the skin) was compared to a less invasive method (EVD placed through a 2.7–3.3-mm twist drill burrhole and fixed to the bone with a bolt system).

**Methods:**

Retrospective observational study in a single-centre setting between 2008 and 2018. EVD placement was assessed using the Kakarla scoring system. We registered postoperative complications, surgery duration and number of attempts to place the EVD.

**Results:**

Two hundred seventy-two patients received an EVD (61 bolt EVDs, 211 standard EVDs) in the study period. Significant differences between the bolt system and the standard method were observed in terms of revision surgeries (8.2% vs. 21.5%, *p* = 0.020), surgery duration (mean 16.5 vs. 28.8 min, 95% CI 7.64, 16.8, *p* < 0.001) and number of attempts to successfully place the first EVD (mean 1.72 ± 1.2 vs. 1.32 ± 0.8, *p* = 0.017). There were no differences in accuracy of placement or complication rates.

**Conclusions:**

The two methods show similar accuracy and postoperative complication rates. Observed differences in both need for revisions and surgery duration favoured the bolt group. Slightly, more attempts were needed to place the initial EVD in the bolt group, perhaps reflecting lower flexibility for angle correction with a twist drill approach.

## Introduction

Placement of an external ventricular drain (EVD) is one of the most common and important surgical procedures in the acute neurosurgical setting. Indications for EVD placement can be increased intracranial pressure (ICP) secondary to for instance subarachnoid haemorrhage (SAH), intracranial or intraventricular haemorrhage (ICH and IVH) and traumatic brain injury (TBI). Despite the use of anatomical landmarks to determine Kocher’s point and our knowledge of estimating the trajectory toward the ipsilateral frontal horn, accurate EVD placement can be challenging. Numerous studies have assessed ways of optimising drain placement [12, 14, 16, 19]. Thus, developing and improving surgical methods to improve accuracy and minimise complications rates continue to be of great interest.

From 2015 and onwards, our department adopted a less invasive method, a so-called bolt system and partly replaced our previous standard technique of using a tunnelated drain. We therefore sought to evaluate whether this new technique provides any clear benefit over an already standardised procedure.

The aim of the present study was to assess and compare the two different surgical methods in terms of accuracy of EVD placement in accordance with the Kakarla scoring system [14]. Furthermore, we wished to review complication rates, and determine whether there were any additional factors indicating superiority of one method over the other.

## Materials and methods

### Data collection

The current study was a retrospective study. We used the Nomesco Classification of Surgical Procedures (NCSP) to identify all patients who had received an EVD at the neurosurgical department at St. Olavs University Hospital, Trondheim, Norway in the period between January 1 2008 and January 1 2019. After extracting these data, three authors reviewed all patient records to identify which of these patients fulfilled the inclusion criteria. One author then reviewed the data and checked for consistency.

The inclusion criteria were as follows: (1) adult patients over the age of 18, who (2) had received an EVD using one of the two surgical methods and (3) were admitted primarily to the aforementioned institution. Following the introduction of the minimally invasive method in 2015, the choice of surgical method used in each case was by default not randomised, but rather determined by the surgeon’s own preference. There were no exclusion criteria in regard to indication for performing the surgery. However, patients receiving an EVD as part of other surgeries, such as tumour resection, aneurysm clipping and endoscopic procedures were excluded.

**Fig. 1:**
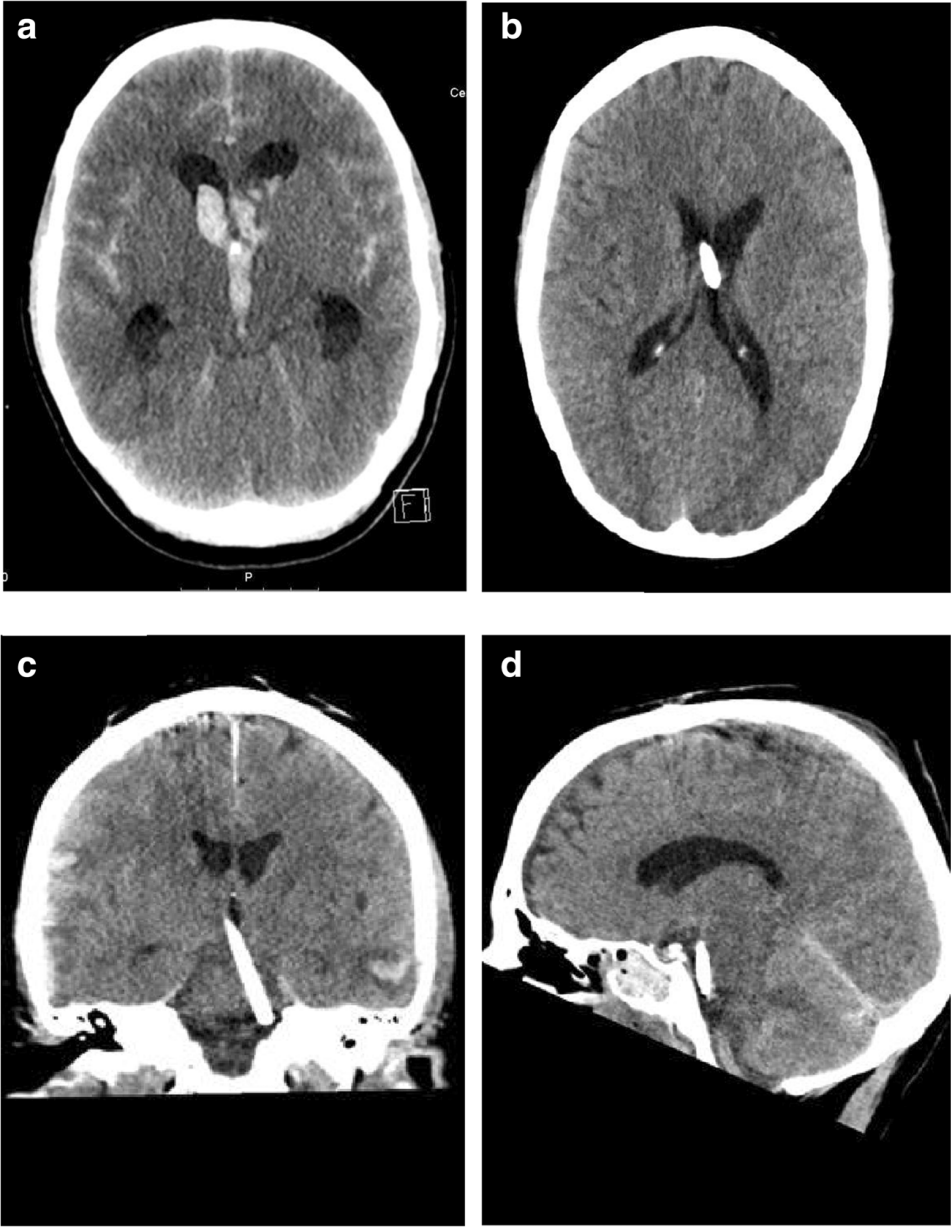
**a–d** Postoperative CT scans. **a** Kakarla score 1; tip of EVD in third ventricle. **b** Kakarla 2; tip of EVD in contralateral ventricle. **c**, **d** Kakarla 3; tip of EVD in basal cisterns-prepontine cistern (this patient underwent revision surgery)

We extracted and recorded baseline data for all patents including gender, age, comorbidities, diagnosis/indication for surgery and characteristics of preoperative imaging (CT or MRI); calculating the Evans index, measuring midline shift if any, assessing the modified Graeb Scale (mGS), Fischer score for aneurysmal SAH and calculating the volume of ICH if any. Furthermore, we extracted data on the characteristics of the EVD procedure itself, including surgery duration, number of passes to place the EVD, (established from the operation notes), frequency of revision, and if revisions were performed, the reasons for the revision(s). We assessed the placement of the EVD by reviewing postoperative imaging using the Kakarla scoring system [14]; Grade 1 means optimal placement in the ipsilateral frontal horn or third ventricle; Grade 2 means a functional placement in the contralateral lateral ventricle or noneloquent cortex; and Grade 3 means suboptimal placement in the eloquent cortex or nontarget cerebrospinal fluid space, with or without functional drainage (Fig. 1).

### Surgical procedure

The two surgical methods we evaluated were as follows (1) standard technique and (2) the bolt system.

In our department, the standard technique entails estimating Kocher’s point using anatomical landmarks (approximately 1–2 cm frontal to the coronal suture and 2–3 cm lateral to the midline), and making a 14-mm burrhole using an automatic trephine. Thereafter, the dura is opened, usually in a cruciate manner, followed by a small corticotomy before the drain is placed. Following cerebrospinal fluid (CSF) response, the drain in tunnelated through a separate skin incision, where the catheter is secured with a suture before the skin is closed with continuous sutures.

The bolt method utilises the same standard preparation to assess Kocher’s point, but instead a 2.7–3.3-mm twist drill burrhole is made using a manual drill. The dura is either lacerated by the drill or opened by using a pointed awl to prick a small hole in the dura. The drain is then placed estimating the trajectory similarly to the standard technique. After CSF response, the drain is then fixed to the skull bone using a bolt system (Spiegelberg).

While the standard method is primarily performed in a neurosurgical operating room (NOR), the less invasive method is more frequently performed bedside in the intensive care unit (ICU). The procedure is usually performed with local anaesthesia, with or without sedation. Navigation or ultrasound is not routinely used for EVD placement in our department, and was not used in the current study. The patients were routinely given one doze of cefalotin 2 g intravenously at the time of surgery. In case of penicillin allergy, clindamycin 600 mg intravenously was used.

### Statistical analysis

All statistical analyses were performed using SPSS, IBM version 25. The 2-sample *t* test was used to compare groups for all continuous variables and data was expressed as means ± standard deviation. The chi-square test or Fisher’s exact test was used for all categorical data and expressed as frequencies with counts and percentages. The *p* value for statistical significance was determined to be 0.05.

## Results

### Patient demographic and characteristics

A total of 272 patients were included in the present study. Two hundred eleven patients received a tunnelated EVD, and 61 patients received a bolt EVD. Table 1 provides a summary of patient characteristics; the baseline characteristics were similar for both groups with the exception of preoperative Evans ratios. The most common diagnosis in both groups was SAH, with approximately 50% in each group. Of all patients presenting with intracranial haemorrhage, intraventricular blood was present in approximately 80% in both groups. For all patients with aneurysmal SAH, a Fischer score was assigned, with similar results in both groups. In case of IVH, the mGS score was calculated for each case, showing similar results in both groups.

**Table 1:** Baseline characteristics

	Tunnelated	Bolt	*p* value
	*N* = 211 (%)	*N* = 61 (%)	
Age, mean (SD)	56.9 (14.6)	58.6 (15.5)	0.429
Gender, male	117 (55.5)	29 (47.5)	0.275
Evans index, mean (SD)^1^	0.32 (0.07)	0.30 (0.05)	*0.036*
Midline shift, mean (SD)	1.05 (2.48)	1.09 (2.89)	0.906
Diagnosis			
SAH	104 (49.3)	32 (52.5)	0.663
IVH	3 (1.4)	4 (6.6)	*0.026*
ICH	42 (19.9)	14 (23.0)	0.604
TBI	23 (10.9)	7 (11.5)	0.900
Meningitis	7 (3.3)	2 (3.3)	0.988
Tumour/malignancy	24 (11.4)	0 (0.0)	*0.006*
Other	8 (3.8)	2 (3.3)	0.851
Comorbidities			
Hypertension	69 (32.7)	22 (36.1)	0.775
Cerebrovascular disease	29 (13.7)	9 (14.8)	0.850
Cardiovascular disease	28 (13.3)	14 (23.0)	0.162
Diabetes	16 (7.6)	4 (6.6)	0.832
Chronic pulmonary disease	24 (11.4)	6 (9.8)	0.814
Tumour/malignancy	20 (9.5)	2 (3.3)	0.251
Haematological disease	4 (1.9)	1 (1.6)	0.857

*p* values marked in italics indicate significance

^1^Confidence interval − 0.039, − 0.001. *SD* standard deviation, *SAH* subarachnoid haemorrhage, *IVH* intraventricular haemorrhage, *ICH* intracranial haemorrhage, *TBI* traumatic brain injury

### Characteristics of the procedure and EVD placement

As seen in Table 2, surgery duration between the two groups differed significantly; mean surgery duration in the tunnelated group was 28.8 ± 16.8 (range 4, 52), versus 16.5 ± 10.1 (range 8, 89) minutes in the bolt group (95% CI 7.64, 16.8, *p* = 0.00). The number of passes in the two groups differed significantly with a mean of 1.32 ± 0.8 and 1.72 ± 1.2 (95% CI − 0.74, 0.08, *p* = 0.017) for the tunnelated and bolt group, respectively. There was a trend towards lower frequencies of Kakarla 1 scores in the tunnelated group compared to the bolt group, not reaching statistical significance (*p* = 0.089), and similarly a higher frequency of Kakarla 2 scores, reaching statistical significance (*p* = 0.043). The two groups had similar Kakarla 3 scores of 12.3% and 12.5% for the tunnelated and bolt group, respectively (*p* = 0.977).

**Table 2:** Characteristics of the surgical procedure

	Tunnelated		Bolt		*p* value
	no/total no		no/total no		
Surgery duration in minutes, mean (SD)	161/211	28.8 (16.8)	59/61	16.5 (10.1)	*0.000*
Number of passes, mean (SD)	142/211	1.32 (0.8)	36/61	1.72 (1.2)	*0.017*
1 pass (%)	118/146	80.8	25/36	69.4	0.136
> 1 pass (%)	28/146	19.2	11/36	30.6	0.136
Placement, *n* (%)					
Kakarla 1	170/211	85 (49.4)	56/61	35 (62.5)	0.089
Kakarla 2		68 (40.0)		14 (25.0)	*0.043*
Kakarla 3		21 (12.3)		7 (12.5)	0.977

*p* values marked in italics indicate significance

*SD* standard deviation

### Surgical complications

There was a statistically significant difference in the frequency of EVDs requiring later revision(s), with 21.4% in the tunnelated group versus 8.2% in the bolt group (*p* = 0.020). The cause of revision surgery was similar in both groups, showing a trend where occlusion and accidental removal were more common in the tunnelated group, however not reaching statistical significance. Postoperative haematoma occurred in a total of 5 (2.7%) patients in the tunnelated group, and 3 patients (5.3%) in the bolt group (*p* = 0.340) (Table 3). The haematomas were all characterised as track haematomas, without clinical significance, nor did any of these patients consequently require surgery. Postoperative meningitis occurred in 15 cases (7.1%) in the tunnelated group and in 1 case (1.6%) in the bolt group (*p* = 0.110).

**Table 3:** Surgical complications

	Tunnelated *N* = 211 (%)	Bolt *N* = 61 (%)	*p* value
Postoperative haematoma	5/186 (2.7)	3/57 (5.3)	0.340
Postoperative meningitis	15 (7.1)	1 (1.6)	0.110
Frequency of revisions	45 (21.3)	5 (8.2)	*0.020*
Cause of revisions			
Misplacement	10 (4.7)	2 (3.3)	0.625
Occlusion	29 (13.7)	2 (3.3)	*0.023*
Accidental removal	13 (6.2)	1 (1.6)	0.159

*p* values marked in italics indicate significance

## Discussion

The current study evaluates 11 years of EVD placement using the standard tunnelated technique with a standard trephine hole versus the newly adopted twist drill and bolt system. The current data indicates that the two methods are similar in terms of accuracy and complication rates. Advantages of the bolt system are the shorter surgery duration and the less frequent need of revision surgery. Hence, the bolt system seems to serve as a valuable tool in our practice with clinically relevant benefits.

We found a significant difference in the duration of surgery, where the use of the bolt system proved an efficient method for placing an EVD, despite the mean number of passes being greater than in the tunnelated group; hence, it seems appropriate to conclude that the surgical method is responsible for the longer duration of surgery and not necessarily the number of passes. Longer surgery duration in the standard tunnelated group is likely due to the more comprehensive task of opening and closing the skin and dura. To our knowledge, no other studies have reviewed and compared these two surgical techniques in terms of surgery duration as a premise for benefit and success. Studies assessing surgery duration and risk of surgical site infection (SSI) in neurosurgical procedures found that longer surgery duration was clearly associated with increased risk of surgical site infections (SSI) [2, 10]. Although surgery duration in the aforementioned studies were generally longer, and occurrence of SSI was not assessed in the current study, it still highlights that duration of surgery does indeed matter, and should be an important factor when reviewing surgical methods and which method to utilise.

A major advantage of the bolt system is the possibility to perform the procedure by the bedside with limited additional personnel. Our study demonstrates that the bolt system could be preferable in patients with acute hydrocephalus in need of quick intervention. One study reviewing the occurrence of complications and accuracy of EVD placement in the OR versus the ICU, favoured placement in the OR, especially in high-risk patients [7]. This is not in line with our data, and may not be relevant, as it did not review the bolt system. A recent review found no convincing evidence that EVD placement outside of the OR presented increased risk in itself [8].

In the current study, we failed to detect differences in the occurrence of postoperative hematoma in the two groups, and the frequency was also considerably lower than previous reports [6, 9, 17, 18, 21–23]. Studies have previously reported rates of close to non-existent to approximately 40% [9, 17, 22]. One radiographic simulation estimated a haemorrhage risk of 19% when reviewing multiple possible trajectories [19]. One study reported a lower frequency of postoperative haematoma in the bolt-group and an overall postoperative haematoma rate of 17.9% [21]. Our findings are more consistent with that of two meta-analyses [1, 4]. Notably, the authors highlighted that in the instances where postoperative CT scans were routinely performed, the rate of postoperative haematoma was much higher [1, 4]. In our study, postoperative CT or MRI scans were available for 186 of the 211 patients in the tunnelated group, and 57 of the 61 patients in the bolt group. Had scans been available for all patients, the rates of postoperative haematoma might have been affected.

One study also reviewed the incidence of haematoma as a consequence of EVD removal, and found a surprisingly high frequency of 22.5% [18]; the scope of occurrence of postoperative haematoma might be greater than previously reported had this been more frequently been reviewed. From a clinical perspective however, the most valuable aspect of any complication is when a complication results in new symptoms and/or require additional surgery, which was not the case in our study for any of the postoperative haematomas. This is similar to previous studies which report clinically significant hematomas of well under 1% [1, 4].

Several studies have assessed possible risk factors for developing postoperative haematomas [17, 20, 22]. .In the current study, the number of attempts to place the EVD was higher in the bolt group; a natural assumption is that increased number of passes used to place the EVD could correlate to the occurrence of postoperative haematoma and perhaps also the type/severity of haematoma. A recent study reported a clear association between number of attempts to place the EVD and increased risk of haemorrhage [18]. We found no such link however. In a multivariate analysis, one study found that age > 75 years was an independent risk factor of postoperative haematoma [22]. Another study found that patients receiving antiplatelet medication within 96 h of EVD placement were 13 times more likely to exhibit new or enlarged intracerebral bleed [20]. One study found increased risk of postoperative haematoma in patients with established cerebrovascular disease [17]. However, evaluating risk factors for the clinically relevant postoperative haematomas represents a greater challenge, as the incidence is so low.

Occurrence of postoperative meningitis was low in both groups, and no differences were found. The definition of catheter-associated infection varies across studies. In the current study, meningitis was defined as positive CSF findings with increased levels of leukocytes and suspected infection either due to further lab findings such as elevated white blood cells (WBC) and/or C-reactive protein (CRP) and/or clinical findings. The difficulties in assessing catheter-induced central nervous system (CNS) infection remain however challenging and have previously been acknowledged [8, 15]. Reports of infection rates have previously varied and reported as high as 30%, but most commonly around 10% [8, 15]. Both groups in our study are well below these numbers. Infection rates can possibly be influenced by the predisposing diagnosis and hospitals’ bacterial flora, as well as irrigation and duration of EVD treatment with potential increased risk with a longer duration, but not necessarily in a linear manner [8, 15]. This was however not assessed in the current study.

Studies have previously examined accuracy of freehand EVD placement, which is the current practice in our department, and have found that there is a considerable amount of inaccuracy regardless of method [11]. One study reported increased accuracy with the freehand technique using the bolt system compared to the tunnelated standard method [3]. In terms of EVD placement, there was only a significant difference in the proportion of patients with a suboptimal placement (Kakarla 2), favouring the tunnelated method. However, seeing as a Kakarla score of 2 usually will still give a functional EVD, this does not seem to be of great clinical relevance. Importantly, the placement of the drain in eloquent areas is similar in both groups (Kakarla 3), and these are the EVDs that most likely will require revision surgery.

An important factor that may have influenced these results is the difference in Evans ratio, favouring the tunnelated group; this may have resulted in improved accuracy in this group, as well as a lower number of attempts needed to place the EVD. Further analyses failed nonetheless to show any specific trends in terms of Evans ratio and number of attempts. It could be speculated however that if the first attempt of placing an EVD fails, the small twist drill hole could be a disadvantage in the flexibility in angle correction in subsequent attempts.

Furthermore, we did not assess whether EVD accuracy correlated with the surgeon’s skill or experience; previous studies have not found significant differences between midlevel practitioners and experienced neurosurgeons in terms of accuracy or complication rates (haemorrhage, infection) [5, 14].

In the literature, data on the difference in rate of revision surgery are conflicting [3, 13, 21]. In the current study, revisions were required more frequently in the tunnelated group. The cause of revision was most often due to occlusion. One reason for occlusion could be the presence of intraventricular blood, with blood clotting the tip of the EVD. The assessment of the IVH in terms of mGS however proved to be similar in both groups. It is difficult to explain why there was a greater occurrence of occlusion in the tunnelated group, but this may have been associated with misplacement; in the tunnelated group, there was a trend towards fewer EVDs in optimal placement compared to the bolt group. One recent study reported that permanent catheter occlusion was however more frequently correlated to small catheter diameter and therapeutic anticoagulation, whilst a non-ideal catheter position was only marginally significant [6].

Limitations of the current study include those inherent of an observational study, and it would be preferable to have conducted a randomised controlled trial to avoid any risk of selection bias. However, we believe there is no significant selection bias in the current study; when reviewing the operation notes, only a minority of surgeons consistently chose to perform the standard method throughout the study period. Most surgeons adopted the bolt method with a gradual increase in use after 2015, resulting in most surgeons choosing to exclusively performing bolt surgery regardless of indication (CT findings etc.,) by the end of 2018. We therefore feel that the choice of method has rather been determined by the availability of the bolt method from 2015 and surgeons own preference, and not patient characteristics such as ventricular size. An RCT would however be useful to further explore the current findings. Additionally, the bolt group was relatively small compared to the tunnelated group. Another limitation was missing data for some patients, such as number of attempts, postoperative imaging, direct references to antibiotic regimes and surgery protocols. Strengths of this study include high external validity, the pragmatic study design and relatively large number of patients. Although three authors contributed to extracting the data, one of the authors reviewed all the collected data and checked for inconsistencies and reassessed any areas of conflict, in this way assuring uniformity in the collected data.

## Conclusions

The standard tunnelated and bolt techniques are similar in regard to accuracy and postoperative complication rates. Advantages of the bolt system are the significantly shorter surgery duration and lower frequency of revision surgery, features that should impact on the decision making in regard to the choice of method utilised. The current study highlights some important factors in regard to method superiority favouring the bolt system with potential implications for surgeons and patients in the acute neurosurgical settings where available time in the operating room can be a limiting factor.
